# Split Body Trials in Systematic Reviews and Meta‐Analyses: A Tutorial

**DOI:** 10.1002/cesm.70052

**Published:** 2025-09-24

**Authors:** Nuala Livingstone, Kerry Dwan, Marty Chaplin

**Affiliations:** ^1^ Cochrane Evidence Production and Methods Directorate London Greater London UK; ^2^ Centre for Reviews and Dissemination University of York York North Yorkshire UK; ^3^ Liverpool Reviews and Implementation Group University of Liverpool Liverpool Merseyside UK

## Abstract

This tutorial focuses on split body trials in the context of a systematic review and meta‐analysis. We will explain what split body trials are, the potential unit of analysis issues they can cause, and how to include data from split body trials in a systematic review.

Split body trials micro learning module

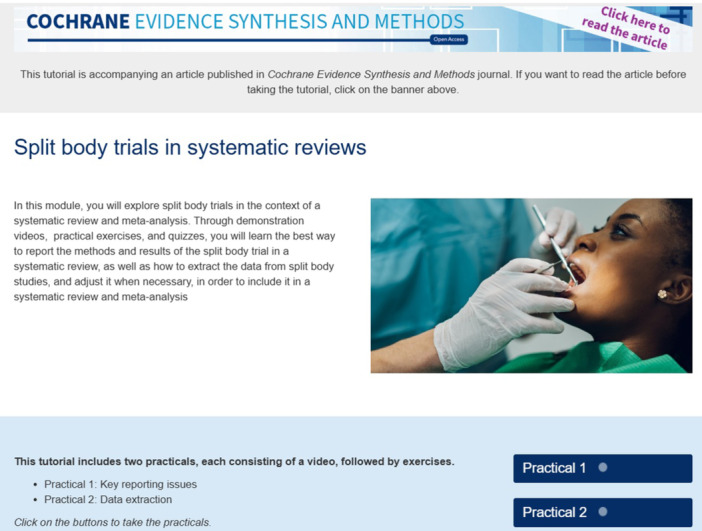

This tutorial focuses on split body trials in the context of a systematic review and meta‐analysis. We will explain what split body trials are, the potential unit of analysis issues they can cause, and how to include data from split body trials in a systematic review.

## 1 What Is a ‘Split Body’ Trial?

A ‘Split Body Trial’ refers to when randomization of interventions takes place within individuals, with different interventions being applied to different body parts. Some common types of this design can be seen in oral health studies making use of a “split mouth” design (e.g., intervention A is applied to the left quadrant of the mouth, and intervention B is applied to the same participant's right quadrant), or eyes and vision studies, making use of a “paired eye” design (e.g., intervention A is applied to a participant's left eye, and intervention B is applied to the same participant's right eye).

This tutorial does not cover trials where individuals are randomised, but multiple body parts within each individual receive the same intervention. Such trials are analysed similarly to cluster‐randomized controlled trials (i.e. each individual is a “cluster” of body parts) for which separate guidance is available [1].

Some trials may have both split body and clustering aspects, i.e. randomization of interventions takes place within individuals, and multiple body parts within each individual receive the same intervention. For example, in a trial of two different topical creams (Cream A and Cream B) for treating psoriasis, the left side of each individual's body is randomized to receive either Cream A or Cream B, and the right side receives the other cream. The outcome measure of interest is measured for multiple psoriasis plaques on each side of the body. Here, the trial employs a split body design, but observations are also clustered, as all the plaques within one side of the body receive the same intervention. Analyses of such trials should account for both the split body aspect of the design (which is the focus of this tutorial) and the clustering aspect of the design (which is the focus of a separate tutorial [1]).

## 2 Why Do We Need to Treat Data From Split Body Trials Differently?

A split body trial design has some similarities to a cross over randomised controlled trial (RCT) design. When the same participant is randomly assigned to receive multiple different interventions simultaneously, (e.g., intervention A is applied to a participant's left eye, and intervention B is applied to the same participant's right eye), then it is important that the subsequent analysis accounts for this pairing of body parts within individuals, similar to how data from cross over studies must also be adjusted to account for the pairing of different intervention periods. Authors of split body trials ought to use appropriate statistical techniques to account for dependency of the data within the individual. Such an approach prevents a unit of analysis error.

## 3 What Is a “Unit‐Of Analysis” Error?

Different body parts from the same individual are likely to respond in a similar way to each other, and therefore observations made for these different body parts cannot be assumed to be independent. A “unit‐of‐analysis” error occurs when the statistical analysis doesn't account for the dependency of measurements within the same participant.

## 4 How Do I Include Data From a Split Body Trial in a Systematic Review?

When including data from a split body trial in a systematic review, review authors should consider whether the intervention could be carried across from one body part to contaminate the control body part. If the risk of contamination is significant, review authors should not include the trial in meta‐analyses. Instead, the trial's results should be summarized narratively, with clear acknowledgement of the limitations regarding contamination.

If the risk of contamination is deemed to be low, and review authors judge that it is appropriate to include the split body trial in meta‐analysis, it is important that the effect estimate and its corresponding confidence interval account for the paired nature of the data.

For continuous outcome data, one appropriate approach is to extract (if available) data that summarise participant‐specific differences, i.e. for each participant, the difference between the measurement taken for the body part that received the experimental intervention, and the measurement taken for the body part that received the control intervention. The mean and standard error of these participant‐specific differences can be included in meta‐analysis using the generic inverse variance method.

Alternatively, if the study authors report a mean difference and either i) a *t*‐statistic from a paired *t*‐test; (ii) a *p*‐value from a paired *t*‐test; or (iii) a confidence interval from a paired analysis, then these values can be used to calculate a standard error that accounts for the paired nature of the data. You can perform the calculation yourself using methods described in the Cochrane Handbook Section 6.5.2.3, or use meta‐analysis software that is able to perform these calculations for you. You would then be able to conduct meta‐analysis using the generic inverse variance method. NB. We have used the term “mean difference” here, which can be interpreted as the difference between the mean of measurements taken for body parts that received the experimental intervention and the mean of measurements taken for body parts that received the control intervention, or as the mean of the participant‐specific differences, as the two values are inherently equal.

If study authors report summary statistics as if the study was a “standard” RCT (i.e. mean and standard deviation for each group), it would be possible to calculate the mean difference required to include data from a split‐body trial in meta‐analysis (by subtracting the control group mean from the intervention group mean). However, it would not be possible to calculate the standard error required for meta‐analysis (which accounts for the paired nature of the data) based on the reported means and standard deviations.

If suitable summary data are not reported by study authors, review authors could calculate these summary data themselves if they have access to individual participant data from the study, or if they are able to extract matched outcome measurements for each individual from graphs presented in the published article.

For other outcomes, analyses are more complex and we advise you to seek guidance from a statistician.

## 5 How Do I Perform Risk of Bias Assessments for Split Body Trials?

Review authors should use the variant of the Cochrane risk‐of‐bias 2 tool [2] for randomized trials that is specific to crossover trials, for which detailed guidance is available [3]. While the tool focuses on crossover trials with two intervention periods rather than two body parts, its principles can be applied to identify potential sources of bias in split body studies. However, instead of focusing on ‘carry‐over effects’ and ‘period effects’, review authors should prioritize evaluating the risk of contamination between the two body sites.

## 6 Common Queries

### 6.1 What If It Is Not Possible to Obtain an Adjusted Effect Estimate?

If it is not possible to extract or calculate an adjusted effect estimate from data reported in the published paper, review authors may be able to obtain this by contacting study authors.

Another option is for review authors to approximate a paired analysis by imputing missing standard deviations (See Cochrane Handbook Section 23.2.7) [4].

As a last resort, review authors may decide to incorporate data from an unpaired analysis of the split body trial. Although this analysis approach is incorrect, it is also conservative, as the trial will receive too little weight in the meta‐analysis, and confidence intervals will be artificially wide. If review authors take this approach, it is important to be aware that clinically important heterogeneity may be obscured by confidence intervals that are too wide. Sensitivity analyses can be used to evaluate whether meta‐analysis results are impacted by the inclusion of data from split body trials that do not account for within‐subject dependency.

Finally, review authors always have the option to exclude results from split body trials from meta‐analyses. Instead, results could be reported in tables and text, with clear acknowledgement of the limitations of the unadjusted data.

### 6.2 Can I Include Split Body Trials and RCTs That Randomise Individuals in the Same Meta‐Analysis?

Yes, although it would be sensible to introduce subgroups to the analysis, so that split body trials and RCTs that randomised individuals are grouped separately on the forest plot. This will improve interpretation of the meta‐analysis results.

## 7 Further Reading and Online Content

Analyses of split body trials should follow the same principles as analyses of crossover trials [5, 6, 7]. The Cochrane Handbook for Systematic Reviews of Interventions [4] provides information on crossover trials in Chapter 23.2.

A CONSORT 2010 extension checklist [8] includes a summary of key methodological features of split body trials, which are referred to as “within person randomized trials”. Lesaffre et al. [9] discuss the split mouth design, specific to dentistry studies, in detail; the concepts covered can be applied to split body trials in general.

**Figure 1:**
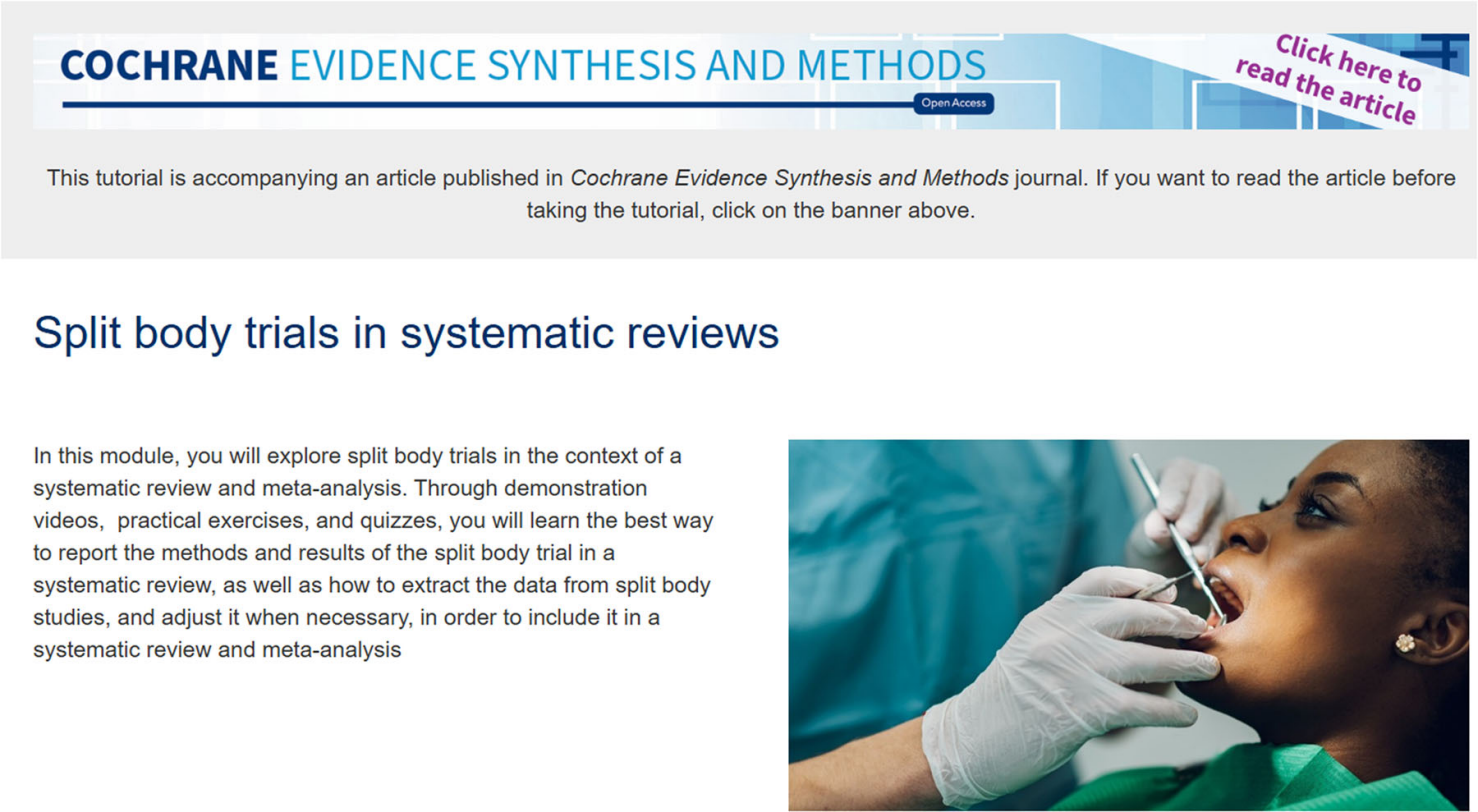
Screenshot of micro learning module.

Cochrane Training have produced a micro learning module [10] to accompany this tutorial. The module contains videos and practical exercises that demonstrate how to include split body trials in systematic reviews and meta‐analyses (Figure 1).

## Author Contributions

**Nuala Livingstone:** conceptualization, writing – original draft, writing – review and editing. **Kerry Dwan:** conceptualization, writing – review and editing. **Marty Chaplin:** writing – original draft, writing – review and editing.

## Conflicts of Interest

Nuala Livingstone is employed by Cochrane. Kery Dwan is Statistical Editor for CESM but had no role in the editorial part of this tutorial. Marty Chaplin declares no conflicts of interest.

## Supporting information

CESM‐Declaration‐of‐Interest FORM.

## Data Availability

Data sharing not applicable to this article as no datasets were generated or analysed during the current study.
